# Image Findings Can Predict Possible Immunoglobulin G4-Related Diseases: A Case Report

**DOI:** 10.7759/cureus.89835

**Published:** 2025-08-11

**Authors:** Kaori Kuriu, Shoji Oura

**Affiliations:** 1 Department of Surgery, Kishiwada Tokushukai Hospital, Kishiwada, JPN

**Keywords:** igg4-related disease, internal high echoes, micro voids, obliterative phlebitis, plasma cells

## Abstract

A 66-year-old man with a history of well-differentiated rectal adenocarcinoma surgery 10 years before had been followed up for his abdominal mass, 1.2 cm in size, just adjacent to the inferior vena cava. The retroperitoneal mass fortunately showed only a nominal growth for more than five years. The retroperitoneal mass, however, showed rapid growth up to 2.2 cm after the squamous lung cancer operation. Ultrasound showed that the mass had internal punctate high echoes and enhanced posterior echoes. Magnetic resonance imaging (MRI) showed low signals on T1-weighted images, low signals with intermingling of faint high signals on T2-weighted images, and mixed high and low signals on diffusion-weighted images (DWIs). Positron emission tomography showed a maximum standardized uptake value of 2.7. These findings led us to judge that the mass was not a possible malignancy. However, the patient’s strong preference for surgical removal of the mass made us treat it by surgical intervention. Postoperative pathological study showed that the lymphatic tissue was surrounded by a thick fibrous capsule and also had a large amount of fibrous components within the lymph node. In addition to the obliterative phlebitis within the fibrous components, numerous IgG4-positive plasma cells on immunostaining led to the diagnosis of IgG4-related disease. The patient recovered uneventfully and has been well without any recurrence for more than four years. Diagnostic physicians should note that IgG4-related disease can have very weak enhancement on CT, low signals both on T2-weighted images and DWIs, and internal punctate high echoes presumably due to the abundant presence of micro-voids in its fibrous components.

## Introduction

The retroperitoneal space has various organs and tissues such as the kidneys, adrenal glands, pancreas, abdominal aorta, inferior vena cava, and lymph nodes. Neoplastic and inflammatory disorders can also develop in these organs and tissues. Tissue sampling, however, is generally more difficult in these retroperitoneal organs and tissues than in hollow organs such as the esophagus, stomach, colon, rectum, and trachea. Image diagnosis, therefore, often plays the mainstay, especially for preoperative diagnosis of retroperitoneal disorders.

Immunoglobulin G4 (IgG4)-related disease generally has an unknown etiology, elevated serum IgG4 levels, IgG4-positive plasma cells, marked fibrosis, a high incidence of concomitant asthma and atopic dermatitis, and a good response to steroid treatment. It is well known that IgG4-related diseases generally make swelling, nodules, or sclerotic lesions, often in the retroperitoneal space in addition to the pituitary gland, thyroid, and salivary glands [1,2].

The retroperitoneal lymph nodes can have metastatic foci of various malignant tumors [3,4]. Swollen retroperitoneal lymph nodes, therefore, need careful differential diagnosis between the primary retroperitoneal disorders, including IgG4-related disease and metastasis from prior malignancies, when the patient has a history of them. Swollen retroperitoneal lymph nodes further need careful image evaluation about the anatomical relationship between them and vital organs such as the abdominal aorta, inferior vena cava, and ureters, when being planned to be surgically treated.

We herein report the correlation between their characteristic images and pathological findings through a case of IgG4-related disease in the retroperitoneal lymph node with prior surgery of the well-differentiated rectal adenocarcinoma.

## Case presentation

**Figure 1:**
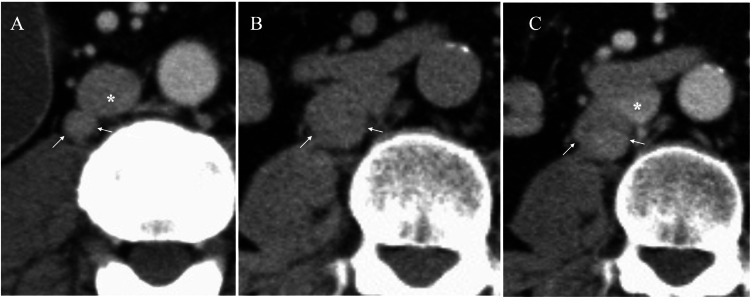
Computed tomography (CT) findings A. CT showed a mass (arrows) located just adjacent to the inferior vena cava (asterisk). B. CT showed enlargement of the mass (arrows). C. CT showed nominal enhancement of the tumor (arrows) with indistinct borders with the inferior vena cava (asterisk).

A 66-year-old man had undergone curative surgery for his well-differentiated rectal adenocarcinoma, followed by capecitabine and oxaliplatin chemotherapy 10 years before. Four years after the operation, computed tomography (CT) revealed a mass, 1.2 cm in size, just adjacent to the inferior vena cava (Figure 1A), which fortunately only showed nominal mass growth during the five-year follow-up period of the rectal cancer. The patient, 11 years after the rectal cancer surgery, underwent right lower lobectomy and lymph node dissection for squamous cell lung cancer with no adjuvant chemotherapy. Follow-up CT for lung cancer, taken one year after lung cancer operation, showed that the retroperitoneal mass rapidly grew up to 2.2cm (Figure 1B, 1C).

**Figure 2:**
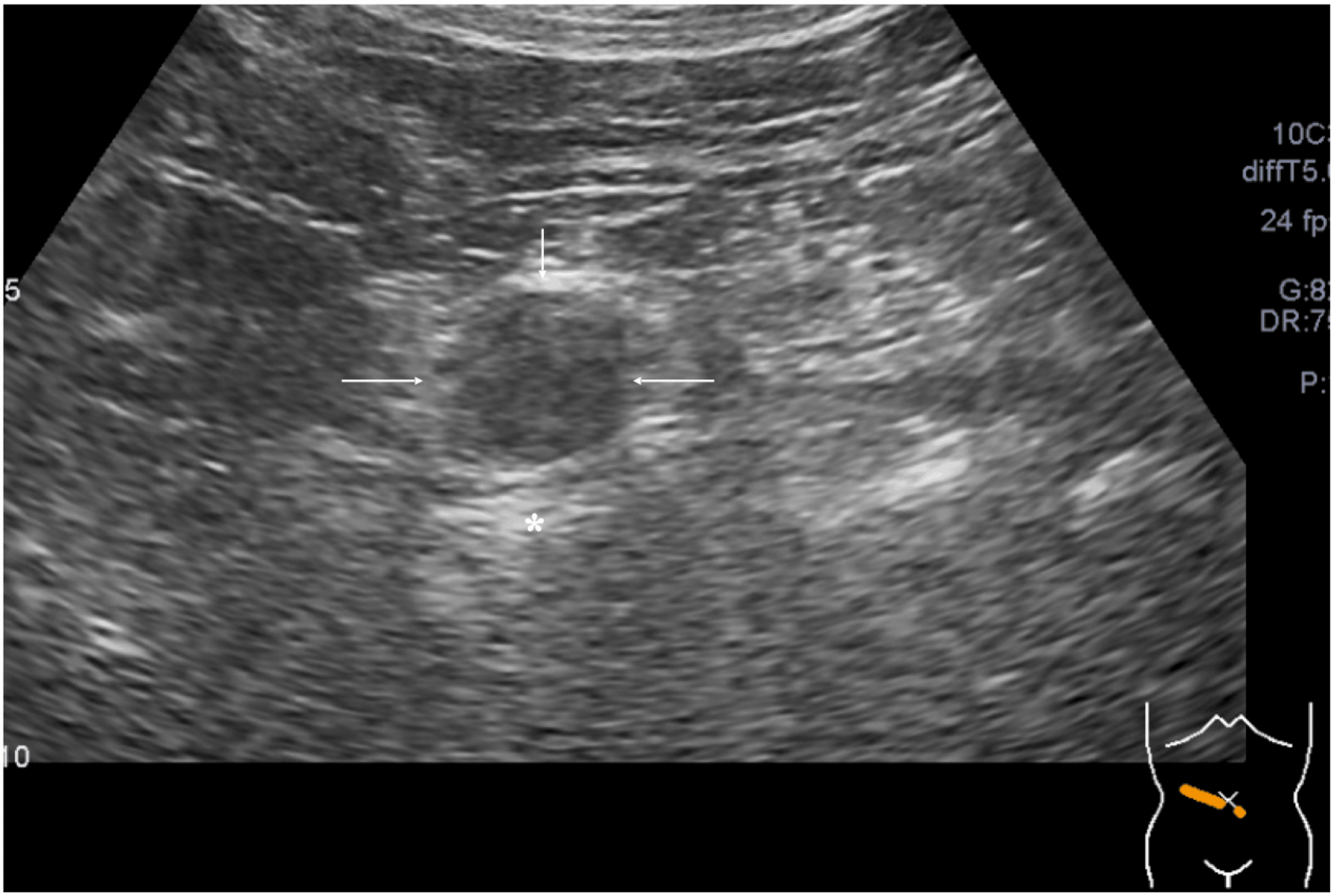
Ultrasound findings Ultrasound showed an oval mass (arrows) with internal punctate high echoes and enhanced posterior echoes (asterisk).

Ultrasound showed that the mass had internal punctate high echoes and enhanced posterior echoes (Figure 2).

**Figure 3:**
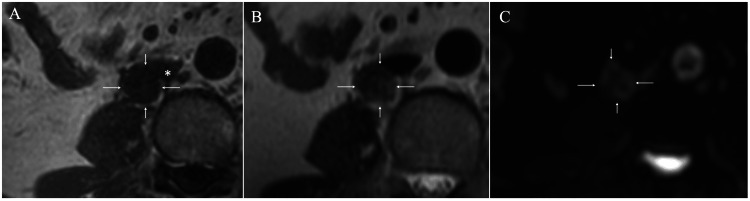
Magnetic resonance imaging (MRI) findings A. MRI of the mass (arrows) showed low signals on T1-weighted images and partially indistinct mass borders adjacent to the inferior vena cava (asterisk). B. MRI of the mass (arrows) showed low signals with faint intra-tumoral high signals on T2-weighted images. C. Diffusion weighted images of the mass (arrows) showed mixed slight high and low signals.

Magnetic resonance imaging (MRI) showed low signals on T1-weighted images (Figure 3A), low signals with interminglement of faint high signals on T2-weighted images (Figure 3B), and mixed high and low signals on diffusion-weighted images (DWI; Figure 3C).

**Figure 4:**
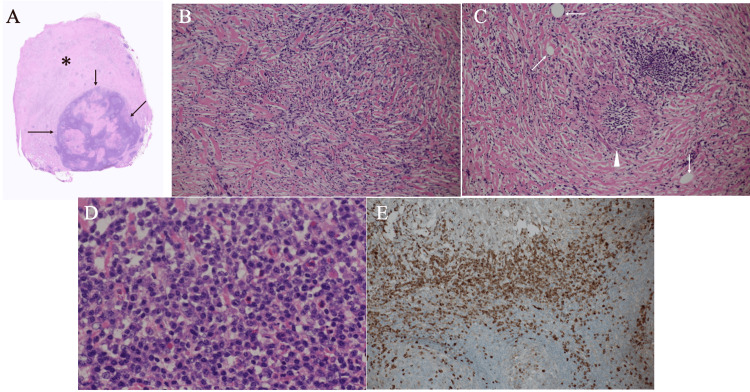
Pathological findings A. Low-magnified view showed the lymph node structures (arrows) and the surrounding thick fibrous layer (asterisk). B. Magnified view showed atypical cells growing in a striform pattern. C. Magnified view showed micro voids (arrows) and an obstructive venule (arrowhead) filled with plasma cells. D. Magnified view showed numerous plasma cells in the lymph node. E. Immunostaining showed numerous IgG4 positive plasma cells (brownish cells).

Positron emission tomography showed a maximum standardized uptake value of 2.7. These findings led us to decide that the mass was not a possible malignancy. Despite our proposal of continued follow-up, the patient’s strong preference for surgical removal of the mass prompted us to treat it by surgical intervention. We could fortunately resect the target mass despite the adhesion between the mass and the inferior vena cava using neither combined vascular resection nor extracorporeal circulation. Postoperative pathological study showed that the lymphatic tissue was surrounded by a thick fibrous capsule containing multiple micro-voids and also had a large amount of fibrous components within it (Figure 4).

In addition to the obliterative phlebitis in the fibrous components, numerous IgG4-positive plasma cells on immunostaining led to the diagnosis of IgG4-related disease. Postoperative laboratory test showed a serum IgG4 level of 19 mg/dl (reference range; 4-108 mg/dL). The patient recovered uneventfully and has been well without any recurrence for more than four years.

## Discussion

Ultrasound showed a well-circumscribed mass with enhanced posterior echoes and internal punctate high echoes against the background low echoes. Internal high echoes generally suggest that masses have some components with different acoustic impedance, e.g., fat, from the surrounding components and/or have ultrasound wave backscattering-inducing structures, including micro-voids, often observed in the papillary or tubular structures [5-8]. Therefore, although the disease-free interval was as long as 10 years in this case, we judged that the prior well-differentiated rectal adenocarcinoma could have developed the metastatic lesion with internal high echoes. However, multiple micro-voids observed in the fibrous components surrounding the lymphatic tissue seemed to actually generate internal punctate high echoes. Further studies are warranted to determine whether IgG4-related diseases can have micro-voids like this case in their fibrous components.

Abundant fibrous components, often observed in IgG4-related diseases, generally make the posterior echoes attenuated [1,2] but failed to attenuate posterior echoes in this case, probably due to the small mass size. Low signals on T2-weighted images highly suggest the abundant presence of pathological components with fewer protons such as fibrous components [9].

Well-differentiated adenocarcinomas have abundant cancer cells and generally show high signals on T2-weighted images, easily negating the possible metastasis of the prior well-differentiated rectal cancer. DWIs further showed slightly high signals at the lymph components but not at the fibrous components.

In addition, CT showed similar image findings of blood flow to those of MRI. In short, slight and delayed enhancement highly suggested nominal blood flow into the mass and was consistent with the abundant presence of fibrous components. IgG4-related disease is a rare disorder that can affect multiple organs [10-12] but naturally can have a limited disease in a single organ only. Absence of its characteristic symptoms can further confound diagnostic physicians. Regardless of the organs affected, however, the presence of abundant fibrosis plays the mainstay in their image diagnosis. It, therefore, is very important for diagnostic physicians to keep in mind that lesions containing abundant fibrous components should include IgG4-related diseases.

Serum IgG4 levels, unfortunately not measured preoperatively in this case, should further contribute to the accurate differential diagnosis of various fibrous component-dominant disorders and can lead to the avoidance of over-treatment. On the other hand, serum IgG4 levels can be within normal ranges when the mass remains small even before surgical removal. Diagnostic therapy using steroids, therefore, should be useful for the presumed IgG4-related small lesions with no elevated serum IgG4 levels.

## Conclusions

IgG4-related disease can have very weak enhancement on CT, low signals both on T2-weighted images and DWI, internal punctate high echoes presumably due to the abundant presence of micro-voids in its fibrous components. Further studies could verify the possible presence of micro-voids in the fibrous components, which can lead to more accurate imaging diagnosis of IgG4-related disease.
